# The Use of Low-Dose Chest Computed Tomography for the Diagnosis and Monitoring of Pulmonary Infections in Patients with Hematologic Malignancies

**DOI:** 10.3390/cancers16010186

**Published:** 2023-12-29

**Authors:** Efthimios Agadakos, Alexandra Zormpala, Nikolaos Zaios, Chrysoula Kapsiocha, Maria N. Gamaletsou, Michael Voulgarelis, Nikolaos V. Sipsas, Lia Angela Moulopoulos, Vassilis Koutoulidis

**Affiliations:** 1 Department of Radiology, General Hospital of Athens Laiko, 11527 Athens, Greece; azormpala@laiko.gr (A.Z.); chkapsiocha@gmail.com (C.K.); 2 First Department of Radiology, School of Medicine, Areteion Hospital, National and Kapodistrian University of Athens, 11528 Athens, Greece; nikzaios@gmail.com (N.Z.); radiology1@med.uoa.gr (L.A.M.); vkoutoulidis@med.uoa.gr (V.K.); 3 Department of Pathophysiology, General Hospital of Athens Laiko, School of Medicine, National and Kapodistrian University of Athens, 11527 Athens, Greece; magama@med.uoa.gr (M.N.G.); mvoulgar@med.uoa.gr (M.V.); nsipsas@med.uoa.gr (N.V.S.)

**Keywords:** low-dose chest CT, standard-dose chest CT, neutropenic patients, hematologic malignancies, radiation dose, CT noise reduction algorithms, diagnostic performance, lung abnormalities

## Abstract

**Simple Summary:**

The research aims to evaluate the diagnostic performance of low-dose chest CT (LDCCT) in detecting pulmonary infections in neutropenic patients with hematologic malignancies. Driven by concerns over radiation exposure in high-risk patients undergoing standard chest CT scans (SDCCT), the research investigates whether LDCCT, with reduced radiation and noise reduction algorithms, matches SDCCT in image quality and diagnostic accuracy. Involving 164 neutropenic patients with 256 CT exams, the study scrutinizes specific radiological criteria linked to pulmonary infections. The researchers analyzed objective parameters (such as image noise and attenuation levels), subjective evaluations (image quality, noise, and artifacts), and diagnostic performance. The findings reveal a 47% reduction in radiation dose with LDCCT but a lower diagnostic performance, especially in detecting consolidation and ground glass opacities. The authors caution against relying solely on LDCCT for initial assessments in patients with hematologic malignancies, emphasizing the importance of further research to optimize diagnostic protocols.

**Abstract:**

The study aimed to assess the image quality and diagnostic performance of low-dose Chest Computed Tomography (LDCCT) in detecting pulmonary infections in patients with hematologic malignancies. A total of 164 neutropenic patients underwent 256 consecutive CT examinations, comparing 149 LDCCT and 107 Standard-Dose Chest CT (SDCCT) between May 2015 and June 2019. LDCCT demonstrated a 47% reduction in radiation dose while maintaining acceptable image noise and quality compared to SDCCT. However, LDCCT exhibited lower sensitivity in detecting consolidation (27.5%) and ground glass opacity (64.4%) compared to SDCCT (45.8% and 82.2%, respectively) with all the respective *p*-values from unadjusted and adjusted for sex, age, and BMI analyses being lower than 0.006 and the corresponding Odds Ratios of detection ranging from 0.30 to 0.34. Similar trends were observed for nodules ≥3 mm and ground glass halo in nodules but were not affected by sex, age and BMI. No significant differences were found for cavitation in nodules, diffuse interlobular septal thickening, pleural effusion, pericardial effusion, and lymphadenopathy. In conclusion, LDCCT achieved substantial dose reduction with satisfactory image quality but showed limitations in detecting specific radiologic findings associated with pulmonary infections in neutropenic patients compared to SDCCT.

## 1. Introduction

Conventional chest radiography has limited sensitivity in detecting lung abnormalities at the early stage of pneumonia [1,2]. Manifestations associated with neutropenia are subtle and difficult to determine with plain chest X-rays [3,4]. As a result, CT of the chest is the imaging method of choice for the prompt detection of radiological signs consistent with pulmonary infections and is frequently performed in patients with hematologic malignancies. Nonetheless, the radiation dose levels of chest CT are substantial and raise radiation concerns for these high-risk patients who are vulnerable to fatal infections triggered by therapy for neutropenia [5]. Moreover, repeated scanning to follow up on pneumonia progression or to monitor patient response to treatment also increases the radiation dose [6,7,8]. It is, therefore, essential that radiation doses are in compliance with the As Low As Reasonably Achievable (ALARA) principle to ensure acceptable diagnostic quality and reasonable image noise levels. The most common dose reduction strategy is tube current reduction and is preferable in Low-Dose CT (LDCCT) protocols [9,10]. Consequently, LDCCT most often yields higher image noise levels and thus to a loss of low-contrast spatial resolution impairing the overall image quality [11]. Even though LDCCT scanning is capable of radiation dose reduction by approximately 1/4 of the standard dose, the radiation-induced cancer risk for doses less than 100 mSv is unpredictable [12,13,14,15]. Since the introduction of iterative reconstruction (IR) algorithms, quantum or statistical image noise can be removed systemically [16]. Today, LDCCT is capable of rendering images of sufficient image quality for the detection of lung abnormalities. In addition, when IR algorithms are implemented, they have the potential to either further reduce image noise or radiation dose depending on the diagnostic requirement [17,18].

To our knowledge, there are no previous studies comparing data sets from low-dose and standard-dose CT examinations of the chest with the use of specific pulmonary infection criteria and statistical-based IR algorithms for adult patients with hematologic malignancies. The aim of our study was to investigate the image quality and the diagnostic performance of LDCCT for the diagnosis and monitoring of pulmonary infections in patients with hematologic malignancies.

## 2. Materials and Methods

The study was compliant with the General Data Protection Regulation (679/2016 EE) and was approved by the Hospital’s Scientific Council and Ethics Committee. Written informed consent was waived due to standard hospital protocol for imaging patients with chest CT for clinical indications including prolonged fever (two to three days), follow-up for interstitial lung disease, unresolved pneumonia, and pulmonary nodules.

### 2.1. Patient Population

All consecutive patients with hematological malignancy referred to our department with the diagnosis of “neutropenic fever”, or fever after chemotherapy, were assessed via their electronic chart for the presence of neutropenia and were included in the study, when the current number of neutrophils was <500 cells/mL, according to current guidelines (7). A statistical power analysis, taking into consideration mainly the incidence of fungal pulmonary infections among hematological patients with fever and neutropenia, defined the minimum number of patients to be included in the study as 150. Consequently, the study sample consisted of 164 neutropenic patients with underlying hematologic malignancies (69 (42%) females and 95 (58%) males with a median age of 64.5 years (IQR 50.5, 71)), contributing data from 256 consecutive non-contrast-enhanced examinations between May 2015 and June 2019 (Table 1). All patients were high-risk neutropenic patients and, at the time of the study, almost all (152/164) were receiving standard antifungal prophylaxis with posaconazole, as per the hospital’s protocols. Regarding antifungal therapy, study patients were treated according to international guidelines (7). More specifically, for neutropenic fever not responding to standard antibiotics, patients were receiving intravenous liposomal amphotericin B or caspofungin; for probable or proven aspergillosis, they were receiving voriconazole, isavuconazole, or liposomal amphotericin B. The patients underwent CT chest examinations prospectively for clinical indications including prolonged fever (two to three days), follow-up for interstitial lung disease, unresolved pneumonia and pulmonary nodules [6,7,8]. The SDCCT protocol was utilized for the initial CT scans, and in the majority of cases, follow-up CT scans adhered to the LDCCT protocol. This has become a standard practice in our department, given the typical requirement for serial CT scans in hematology patients.

### 2.2. CT Scanning Parameters and Data Reconstruction

Data acquisition was performed on a 128 detector array MultiDetection CT system (MDCT) Siemens Somatom^®^ Definition AS+ 128, (Siemens Healthineers, Enlargen, Germany) in the CT Unit of the Medical Imaging Department of the General Hospital of Athens “Laiko”, Greece. The SDCCT protocol selected was the standard protocol used for routine CT chest examinations. The exposure factors were determined according to individual patient size with the use of Automatic Exposure Control (AEC), mA modulation: CAREDose4D™, kV modulation: CARE kV™ (Siemens Healthineers, Enlargen, Germany) corresponding to a variable (Computed Tomography Dose Index volume) CTDIvol: 3.48 mGy (median value). For the LDCCT protocol, the exposure factors were constant: tube voltage: 100 kV; effective current—time product: 40mAs corresponding to a fixed CTDIvol: 1.58 mGy [17,19]. Both protocols generated spiral acquisitions with: slice collimation 0.5 mm; detector configuration 128 × 0.625 mm; pitch ratio: 1.2; rotation time: 0.5 s. Raw data were subsequently reconstructed by applying noise reduction Sinogram Affirmed Iterative Reconstruction Algorithms (SAFIRE^TM^S3-strength:3-Siemens Healthineers, Enlargen, Germany) at a slice thickness of 1 mm using high-resolution lung and standard smooth mediastinal kernels for lung and mediastinal windows, respectively [20]. Image data were sent to the hospital’s picture archiving and communication system (PACS) and to two diagnostic workstations SyngoVia ™ (Siemens Healthineers, Enlargen, Germany). They were displayed on EIZO^TM^ (I MICRO Corporation, Athens, Greece) monitors (matrix: 1536 × 2048; grayscale: 8 bit) using the following viewing parameters: DFOV: 280 mm; image matrix 512 × 512; lung window: WL −700 to −600 HU/WW 1500 HU; mediastinum window: WL 50 HU/WW 350 HU [21]. All data sets from 256 consecutive examinations were used to perform objective analysis, subjective analysis, and diagnostic evaluation of images. The scanning protocols implemented in the study are summarized in the following table (Table 2).

### 2.3. Radiation Dose Measurements-Dose Metrics

The effective dose in milliSievert (mSv) was calculated by multiplying the Dose Length Product (DLP) as recorded on the CT patient dose report upon completion of each examination and the conversion coefficient (k = 0.014 mSv/mGycm) [22]. In general, the SDCCT protocols are consistent with local Diagnostic Reference Levels (DRLs), which are set below the recommended national DRLs [23].

### 2.4. Objective Analysis

The mean attenuation levels (density) and average image noise (standard deviation (SD) of the region of interest (ROI)) were measured in Hounsfield Units (HU). Circular ROIs (surface area: 25–30 mm^2^) were drawn on 1.0mm axial lung images at the level at the aortic arch on 3 anatomical areas: within the tracheal lumen and within the subcutaneous fat on the right and left anterior thoracic walls and on the background. Examinations with poor image quality due to intense artifacts produced by pacemakers or patient arms positioning were excluded. As both the signal-to-noise ratio (SNR) and the contrast-to-noise ratio (CNR) determine image quality, they were calculated using the following equations: [24,25,26].

1. SNR = Density of ROI_A_/SD of ROI_A_
2. CNR = Density of (ROI_A_ − ROI_B_)/SD_B_

Following measurements, all images with ROIs were saved on the patients’ image data sets and transferred to the hospital’s PACS and local diagnostic workstations for future reference and viewing.

### 2.5. Subjective Analysis

Virtual Grading Analysis (VGA) was performed to evaluate the subjective image quality. Axial lung images were reviewed on identical diagnostic workstation EIZO^TM^ monitors ((I MICRO Corporation, Athens, Greece) by two radiographers (E.A, C.K.) with over 20 years of experience in CT. Three image components, image noise, image quality, and occurrence of artifacts (streak, beam hardening and photon starvation artifacts), were rated separately using a 3-point scoring scale for each component, as described in Table 3.

#### 2.5.1. Diagnostic Evaluation

Image analysis was performed in consensus reading by two radiologists (V.K., A.Z.) with over 20 years of experience in CT blinded to the clinical data of the patients. In order to assess the diagnostic performance, data sets obtained from LDCCT and SDCCT protocols were reviewed using the following nine key radiologic findings for assessing pulmonary infection, classified as “present” and “absent” (modified from Patsios D. et al.) [3]:

1. Consolidation
2. Ground Glass Opacity
3. Nodules (≥3 mm)
4. Cavitation in nodule(s)
5. Ground Glass halo in nodule(s)
6. Pericardial effusion
7. Diffuse Interlobular septal thickening
8. Pleural effusion
9. Lymphadenopathy

#### 2.5.2. Statistical Analysis

Demographic and somatometric characteristics of study participants at the first examination were summarized by protocol and overall, using either absolute (N) and relative (%) frequencies for categorical variables or median and interquartile ranges (IQR) for continuous variables. Comparisons between protocols were based on standard procedures (i.e., exact tests for categorical variables and Mann–Whitney U-tests). Descriptive statistics for the aforementioned characteristics were also given for the full sample of examinations. In this case, comparisons were based on appropriate models for clustered data (mixed-effects logistic regression for sex and median regression for clustered data for age and BMI), as each patient may have contributed more than one measurement due to multiple examinations. Similar procedures were used for description and between protocol comparisons of objective analysis, subjective analysis, and diagnostic evaluation. More specifically, medians and IQRs were used for description, and median regression for clustered data was used for between protocol comparisons of all objective analysis results except for one result (Mean Image Noise). The normality of its distribution enabled the use of parametric methods (mean and Standard Deviation-SD for description and mixed linear models for comparisons). All other analyses’ results were categorical, thus absolute (N) and relative (%) frequencies were used for description and mixed logistic (or ordinal logistic) regression models were used for between protocol comparisons. All model-based comparisons were derived through univariable (unadjusted) and multivariable models for adjustment for potential confounding effects of age, sex, and BMI. Interobserver agreements were assessed using the overall percentage of concordance and the relevant agreement statistic (Cohen’s kappa), whereas results were summarized graphically. All analyses were performed using Stata version 15 (StataCorp., College Station, TX, USA). p-values less than 0.05 were considered to indicate statistical significance.

## 3. Results

### 3.1. Description of Study Sample

The study sample consisted of 164 patients who contributed data from 256 examinations. Demographic and somatometric characteristics of the study participants are described in Table 4 and Table 5. As shown in Table 4, 58% of the patients were male, and at the first examination, the median age was 64.5 years and the median BMI was 23.5 kg/m^2^. Most of the study participants (67.7%) had only one examination, and the remaining 32.3% had two or more examinations. Differences in the distribution of these characteristics between the two protocols were small and mostly non-statistically significant. The only statistically significant difference was observed for BMI at the first examination (median 24 versus 23 kg/m^2^ in the low dose versus the standard dose protocol; *p* = 0.002), but this difference ceased to be significant when all examinations were considered (*p* = 0.118).

### 3.2. Effective Dose

The vast majority of the examinations were chest CT scans (76.6%), followed by chest and abdomen CT scans (12.1%), while the remaining examinations included chest and neck or chest and head CT scans (11.3%). The median (IQR) effective dose was 0.71 mSv (0.67, 0.76) in the low-dose protocol compared to 1.50mSv (1.19, 2.30) in the standard-dose protocol, with the difference being statistically significant (*p* < 0.001). Adjusting for sex, age, and BMI, the estimated difference remained practically unchanged [−0.81 (95% CI: −0.97, −0.65; *p* < 0.001)].

### 3.3. Objective Analysis

**Figure 1:**
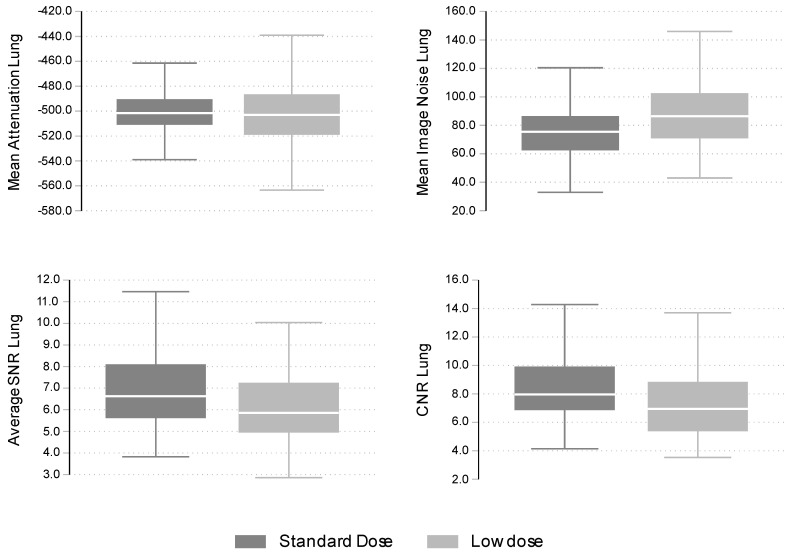
Distribution (boxplots) of objective analysis values by protocol.

Results from the objective analysis by protocol are presented graphically in Figure 1. As shown in this figure, there was no significant difference between the two protocols in the Mean Attenuation levels. The median level of Mean Image Noise was slightly higher, whereas Average SNR and CNR were slightly lower in the low-dose compared to the standard-dose protocol. Quantitative results presented in Table 6 and Table 7 show that differences in Mean Attenuation levels were negligible and non-statistically significant. Conversely, differences in Mean Image Noise were statistically significant (adjusted estimate 12.5; 95% CI: 7.1, 18.0; *p* < 0.001), verifying the higher levels observed in the low-dose protocol (Figure 1). Differences in Average SNR and CNR were of smaller magnitude and statistically significant in univariable analyses but became non-statistically significant when adjusted for sex, age, and BMI (*p*-values 0.046 and 0.083, respectively).

### 3.4. Subjective Analysis

**Figure 2:**
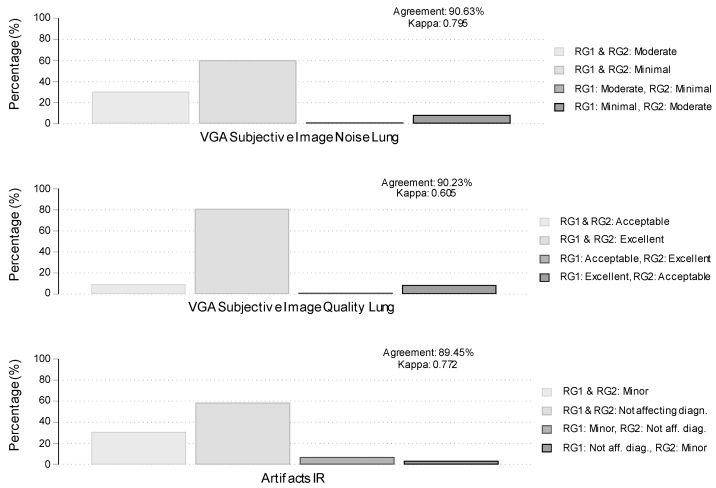
Agreement between examiner #1 (RG1-Radiographer) and examiner #2 (RG2-Radiographer) on subjective analysis. Bars represent percentages of concordance (light gray) or discordance (dark gray) cases. Agreement is the percentage of overall observed agreement, and Kappa is Cohen’s coefficient of agreement.

Results from the comparison between the two examiners regarding subjective analysis results are graphically presented and summarized in Figure 2. The two examiners agreed in approximately 90.63%and 90.23% of the cases regarding their characterizations of Image Noise and Image Quality, respectively. The percentage of concordance decreased to 89.45% regarding their characterizations of Artifacts IR. The corresponding kappa coefficients of agreement were 0.795, 0.605, and 0.772, suggesting very good (Image Noise, Image Quality, and Artifacts IR) agreement. As shown in Figure 2, discordances in characterizations of Image Noise and Image Quality were mainly cases where examiner #1 was giving more favorable characterizations than examiner #2 (21/256; 8.2% for Image Quality and 22/256; 8.6% for Image Noise). Subjective analysis results (by examiner #2) by protocol are summarized and compared between the two protocols in Table 8 and Table 9. Differences were statistically significant (*p* < 0.001 in both unadjusted and adjusted for sex, age, and BMI analyses) for Image Noise. The proportion of cases with “minimal” Image Noise was 45.0% in the low-dose protocol compared to 84.1% in the routine protocol. Regarding Image Quality, examiner #2 characterized as “excellent” 79.2% of the cases in the low-dose protocol compared to 86.0%in the standard-dose protocol, but this difference was non-statistically significant in both unadjusted (*p* = 0.168) and adjusted for sex, age, and BMI (*p* = 0.222) analyses. Finally, results on Artifacts were in favor of the standard-dose protocol with the proportion of cases characterized as “Not affecting diagnosis” being 76.6% compared to 57.7% in the low-dose protocol. This difference was statistically significant in both unadjusted (*p* = 0.002) and adjusted for sex, age, and BMI (*p* = 0.001) analyses.

### 3.5. Evaluation of Diagnostic Performance

**Figure 3:**
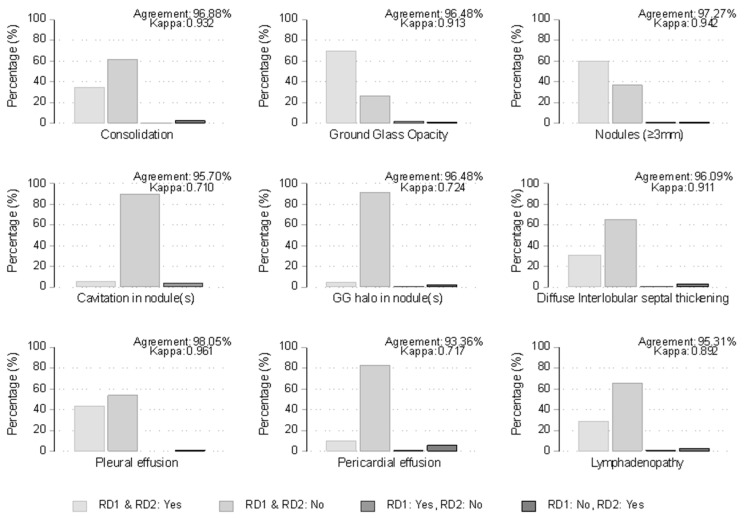
Agreement between examiner #1 (RD1-Radiologist MD) and examiner #2 (RD2) on radiologic findings. Bars represent percentages of concordance (light gray) or discordance (dark gray) cases. Agreement is the percentage of overall observed agreement, and Kappa is Cohen’s coefficient of agreement.

Results from the comparison between the two examiners, regarding radiologic findings associated with pulmonary infections, are graphically presented and summarized in Figure 3. The percentage of agreement between the two examiners ranged from 93% (Pericardial effusion) to 98% (Pleural effusion). As shown in Figure 3, the degree of agreement as estimated by the kappa coefficient was excellent for Consolidation (0.932), Ground Glass Opacity (0.913), Nodules (≥3 mm) (0.942), Diffuse Interlobular septal thickening (0.911), Pleural effusion (0.961), and Lymphadenopathy (0.892), while it was very good for Cavitation in nodule(s) (0.710), Ground Glass halo in nodule (0.724) and Pericardial effusion (0.717). Results by examiner #1 are summarized and compared between the two protocols in Table 10 and Table 11. Differences between the two protocols in the percentage of detection of Cavitation in nodule(s), Diffuse Interlobular septal thickening, Pleural effusion, Pericardial effusion, and Lymphadenopathy were small and statistically non-significant. However, the percentage of detection of Consolidation and Ground Glass Opacity in the low-dose protocol was significantly lower (27.5% and 64.4%, respectively) compared to the standard-dose protocol (45.8% and 82.2%, respectively) with all the respective *p*-values from unadjusted and adjusted for sex, age, and BMI analyses being lower than 0.006 and the corresponding Odds Ratios of detection ranging from 0.30 to 0.34. Similar trends were observed for Nodules ≥3 mm (55.0% in low-dose versus 70.1% in standard-dose protocol) and GC halo in nodule(s) (3.4% in low-dose versus 9.4% in standard-dose protocol), but the corresponding *p*-values were above the nominal level (0.056 and 0.063 from univariable and multivariable analysis, respectively, for Nodules ≥3 mm and 0.062, and 0.114 from univariable and multivariable analysis, respectively, for GC halo in nodules).

## 4. Discussion

Pulmonary infections, especially invasive mold lung infections, including invasive pulmonary aspergillosis and pulmonary mucormycosis, are a significant cause of morbidity and mortality among neutropenic patients with hematological malignancies, and/or hematopoietic stem cell transplantation [27]. Early diagnosis and timely initiation of an appropriate antifungal therapy are of paramount importance, as diagnostic delays are associated with increased mortality [28]. Neutropenia blunts the immune response and the inflammatory process, making diagnostic modalities, such as chest X-ray, ineffective due to low sensitivity [7]. The modern approach to the neutropenic patient with fever is the diagnostic-driven or pre-emptive approach, when the decision to start antifungal therapy is not based solely on the presence of fever not responding to antibiotics but on diagnostic modalities including serial screening of serum galactomannan, aspergillus PCR and serial high-resolution CT scans, on demand [29]. Pioneering work from von Eiff M. et al. and Caillot D. et al. has shown that regular chest CT scanning in febrile neutropenic patients with invasive pulmonary aspergillosis (IPA) can significantly reduce the overall mortality rate by 50% [7,30,31].

The high diagnostic value of high-resolution CT scans in patients with hematological malignancies resulted in an increased frequency of CT examinations, which has tripled over the past 15 years, contributing to almost 60% of the collective radiation dose from medical exposures [32]. Therefore, concerns have been raised about the frequent irradiation of these vulnerable patients, as it can lead to long-term complications whose effects remain unknown [11,26]. In response, international radiation protection authorities have launched dose reduction campaigns, particularly focusing on CT and interventional imaging [33,34].

In recent years, vendors have focused on developing CT systems that incorporate noise reduction algorithms to optimize patient radiation dose while maintaining diagnostic value. These algorithms have been designed to minimize the impact on diagnostic accuracy, ensuring that the quality of the images remains high despite the reduced radiation dose. This development has been ongoing since 2011, with continuous improvements aimed at achieving a balance between dose reduction and preserving diagnostic value in CT imaging [35]. These algorithms systematically remove image noise and are commonly used in ≤1 mm CT imaging to mitigate artifacts (streak, beam hardening, and photon starvation) caused by the dense shoulder girdle [36]. However, the implementation of IR algorithms in lung CT examinations, especially for immunocompromised patients, is a subject of debate. Indistinct findings such as interstitial disease, small nodules, and ground glass opacities (GGO) are best visualized with higher spatial resolution and edge enhancement [37].

It is widely recognized that high-resolution kernels used for lung CT examinations increase image noise [38,39,40]. When combined with thinner slices (<1 mm) and low mAs settings of LDCCT (40 mAs), the reduced radiation dose can further increase image noise and degrade overall image quality. IR algorithms have been shown to reduce image noise, but they exhibit low-contrast detectability similar to Filtered Back Projection (FBP) with higher radiation exposure reductions (>30%) with sufficient noise reduction impartial to image pixelization (blocky appearance) [19,41].

In this work, we reduced the radiation dose of CT scans, and we assessed the diagnostic performance of low-dose CT scans in neutropenic patients with hematological malignancies. We modified the most common dose reduction parameter, the effective tube current, while keeping the reconstruction and image presentation parameters consistent with standard-dose chest CT (SDCCT). Both protocols employed IR algorithms (SAFIRE^TM^) unlike previous studies that focused on comparisons between low-dose chest CT (LDCCT) and chest radiography or between different CT techniques [3,18,19,42,43,44]. Alternatively, our study integrated SAFIRE^TM^ S3 with the strength set at level—S3 (strength: S1–S5) to produce images.

**Figure 4:**
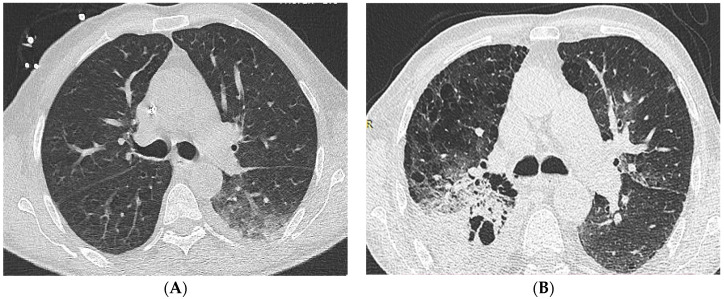
1.0 mm axial CT images demonstrating important pulmonary infection-specific findings i.e., Consolidation, Ground Glass Opacity, and Nodules (≥3 mm) on patients who presented with febrile neutropenia and underlying acute myeloid leukemia; (**A**): SDCCT SAFIRE^TM^ S3 (Deff = 1.06 mSv; SNR = 6.49; CNR = 8.46) on 54 y.o. male (BMI:20); (**B**): LDCCT SAFIRE^TM^ S3 (Deff = 0.74 mSv; SNR = 3.95; CNR = 4.07) on 77 y.o. male (BMI:21).

We found that LDCCT achieved a dose reduction of 47% with satisfactory image quality and acceptable image noise compared to SDCCT (Figure 4). However, the diagnostic performance of LDCCT was lower, underestimating significant radiologic findings associated with pulmonary infections in neutropenic patients, especially consolidation and GGO. LDCCT detected consolidation and GGO in less than 1/3 of cases compared to SDCCT. The detection rate for consolidation and ground glass opacity was influenced by sex, age, and BMI. On the other hand, the diagnostic performance was similar between the two protocols for other radiologic findings such as cavitation in nodules, diffuse interlobular septal thickening, pleural effusion, pericardial effusion, and lymphadenopathy.

A previous study by Hae et al. concluded that ultra-low-dose CT (ULDCT) (Deff: 0.60mSv ± 0.15) with FBP provides acceptable image quality and 63.6% sensitivity for diagnosing pulmonary infections in febrile neutropenic and hematologic malignancy patients. However, the final diagnosis was verified through additional clinical information, laboratory findings, and follow-up chest X-rays [19]. Another study reported that unenhanced LDCCT with IR generated images of improved quality and reduced image noise compared to FBP. They suggested that lesion conspicuity is greatly improved with increasing ASIR strength [43].

Kubo et al. demonstrated that low-dose chest CT (LDCCT) using 50 mAs (effective dose: 3.57 mSv) is effective in detecting various pulmonary abnormalities, including emphysema, ground glass opacities (GGO), reticular opacity, micronodules, bronchiectasis, honeycomb, and nodules larger than 5 mm. In comparison, standard-dose chest CT (SDCCT) using 150 mAs (effective dose: 10.7 mSv) showed similar diagnostic capabilities but with a threefold higher radiation dose. Despite the higher dose in their LDCCT, our study enabled a higher GGO sensitivity at 64% compared to their 49%. Kubo et al. suggested the need for further research on the accurate classification of interstitial pneumonia in patients with a high prevalence of interstitial lung disease [45].

Another study supported the potential of up to 65% dose reduction using SAFIRE^TM^-based chest CT image reconstruction. This approach resulted in images with reduced image noise by 31%–59% and provided good diagnostic confidence compared to conventional chest CT (SDCCT) with filtered back projection (FBP). Although the investigators highlighted the superiority of SAFIRE^TM^ algorithms over FBP, they concluded that these algorithms may not be directly applicable for lung nodule follow-up, lung cancer screening, and bronchiectasis evaluation, where low-dose FBP protocols offer improved visibility of small anatomical structures [19].

Although CT has been the cornerstone for early diagnosis of pulmonary fungal infections among patients with hematological malignancies, biomarkers play a pivotal role as well. A recent prospective study has shown that a preemptive antifungal strategy including twice weekly serum galactomannan screening and CT scan on demand is safe and effective. In addition, this strategy is not associated with an increased risk of invasive fungal infection, and reduces greatly the use of antifungals [29]. Similarly, Picardi M et al., have shown in a retrospective study that among high-risk patients receiving prophylaxis with posaconazole the application of serial serum beta D-Glucan tests and an aggressive strategy of early chest CT scans allows early diagnosis of breakthrough pulmonary aspergillosis, even before the appearance of halo sign and serum galactomannan increase [46].

The limitations of our study include a lack of follow-up on patient progress and the effect of LDCCT on prognosis and survival rate, compared to SDCCT. Additionally, both data sets were obtained using only a single IR algorithm strength setting, specifically SAFIRE^TM^ S3 as incorporated in the departmental protocol for standard-dose chest CT (SDCCT). However, Kalra et al. proposed that using a higher strength setting, such as SAFIRE^TM^ S4, could provide images of acceptable diagnostic quality despite the pixelized appearance [20]. This limitation might have impacted the ability to fully assess the diagnostic performance of low-dose chest CT (LDCCT) in detecting significant pulmonary infection findings, such as consolidation and ground glass opacification in the lungs of patients with hematologic malignancies. Another limitation is that the effect of antifungal therapy on the findings of follow-up LDCCT compared to initial SDCCT was not taken into account. The differences in diagnostic ability might be partly due to the effect of a successful antifungal therapy. However, radiological findings of pulmonary fungal infections, especially nodules, do not abate so quickly after the initiation of antifungal therapy, as they might persist for several weeks.

Further research is advocated, whereby a minimal increase in mAs compared to LDCCT while maintaining a submSv effective radiation dose or a modification in SAFIRE^TM^ strength, may facilitate the detection of pulmonary infection-specific radiologic findings in neutropenic patients with underlying hematologic conditions.

## 5. Conclusions

In conclusion, the results of our study suggest that although LDCCT image quality and image noise are comparable to SDCCT, it underestimates important radiologic findings associated with pulmonary infection such as consolidation and ground glass opacities. The use of LDCCT may not detect early signs of infection and delay the beginning of treatment of pulmonary infections, and therefore it should not be used as the first CT study on patients with hematologic malignancies. However, despite its lower diagnostic ability for specific pulmonary pathology, we think the LDCCT protocol could serve as an adjunctive study when initial SDCCT defines findings consistent with pulmonary infection.

## Figures and Tables

**Table 1:** Hematologic malignancies of patients included in the study.

Hematologic Malignancy	Total Number of Patients *n* = 164	Total Number of Examinations *n* = 256
Acute myeloid leukemia	125	196
Acute lymphoblastic leukemia	23	43
Hodgkin lymphoma	9	9
Non Hodgkin lymphoma	7	8

**Table 2:** Protocol parameters: SDCCT vs. LDCCT.

Standard-Dose Protocol vs. Low-Dose Protocol		
CT Protocol	Standard Dose (SDCCT)	Low Dose (LDCCT)
Exposure factors		
kV	CARE kV™	100
mAs_eff_	CAREDose4D™	40
Rotation time	0.5 s	0.5 s
Acquisition Parameters		
Slice collimation	0.5 mm	0.5 mm
Pitch ratio	1.2	1.2
Reconstruction Parameters		
Slice thickness	1.0 mm	1.0 mm
Algorithm	SAFIRE™	SAFIRE™
Strength (1–5)	3	3
Radiation Dose Descriptors		
*CTDIvol*	Variable *(AEC modulation depending on patient size)*	1.58 mGy

**Table 3:** Subjective image analysis with Virtual Grading of image quality components.

Image Quality Component	Score	Description
Subjective Image Noise		
	2: Minimal	-negligible noise levels not affecting diagnostic accuracy
	1: Moderate	-tolerable noise levels not affecting diagnostic accuracy
	0: High	-increased image noise, grainy image compromising diagnostic accuracy.
Subjective Image Quality		
	2: Excellent	-clearly and well-defined anatomic details, increased diagnostic accuracy
	1: Acceptable	-adequately defined anatomic details, not affecting diagnostic accuracy
	0: Poor	-poorly defined anatomic details, compromising diagnostic accuracy
Artifacts		
	2: Not affecting	-negligible artifact occurrence not affecting diagnostic accuracy
	1: Minor	-tolerable artifact occurrence not affecting diagnostic accuracy
	0: Major	-increased occurrence of artifacts compromising diagnostic accuracy.

**Table 4:** Demographic and somatometric characteristics of study participants by protocol at first examination by protocol.

Variable	Standard Dose	Low Dose	Total	*p*-Value
	*n* = 80 (48.78%)	*n* = 84 (51.22%)	*n* = 164 (100%)	
Sex				0.875
*– Female*	33 (41.25%)	36 (42.86%)	69 (42.07%)	
*– Male*	47 (58.75%)	48 (57.14%)	95 (57.93%)	
Age (in groups—years)				0.854
*– 18–29*	5 (6.25%)	4 (4.76%)	9 (5.49%)	
*– 30–39*	10 (12.50%)	8 (9.52%)	18 (10.98%)	
*– 40–49*	4 (5.00%)	9 (10.71%)	13 (7.93%)	
*– 50–59*	13 (16.25%)	11 (13.10%)	24 (14.63%)	
*– 60–69*	23 (28.75%)	25 (29.76%)	48 (29.27%)	
*– 70–79*	19 (23.75%)	19 (22.62%)	38 (23.17%)	
*– 80+*	6 (7.50%)	8 (9.52%)	14 (8.54%)	
Age (years)—*Median (IQR)*	64.0 (50.0, 70.0)	67.0 (51.0, 71.0)	64.5 (50.0, 71.0)	0.381
BMI WHO categories				0.011
*– Underweight*	1 (1.25%)	2 (2.38%)	3 (1.83%)	
*– Normal*	60 (75.00%)	43 (51.19%)	103 (62.80%)	
*– Overweight*	17 (21.25%)	33 (39.29%)	50 (30.49%)	
*– Obese*	2 (2.50%)	6 (7.14%)	8 (4.88%)	
BMI (kg/m^2^)—*Median (IQR)*	23.0 (21.0, 24.0)	24.0 (22.0, 26.0)	23.5 (21.0, 25.0)	0.002
Number of examinations/patient				0.058
*– 1*	57 (71.25%)	54 (64.29%)	111 (67.68%)	
*– 2*	18 (22.50%)	16 (19.05%)	34 (20.73%)	
*– 3*	1 (1.25%)	10 (11.90%)	11 (6.71%)	
*– 4+*	4 (5.00%)	4 (4.76%)	8 (4.88%)	
Number of examinations/patient—*Mean (SD)*	1.4 (0.8)	1.7 (1.4)	1.6 (1.2)	0.217

**Table 5:** Demographic and somatometric characteristics of study participants (multiple examinations) by protocol.

Variable	Standard Dose	Low Dose	Overall	*p*-Value *
	*n* = 107 (41.80%)	*n* = 149 (58.20%)	*n* = 256 (100%)	
Sex				0.704
*– Female*	48 (44.86%)	71 (47.65%)	119 (46.48%)	
*– Male*	59 (55.14%)	78 (52.35%)	137 (53.52%)	
Age (years)—*Median (IQR)*	62.0 (40.0, 70.0)	64.0 (41.0, 71.0)	63.5 (40.5, 70.0)	0.462
BMI (Kg/m^2^)—*Median (IQR)*	23.0 (20.0, 24.0)	24.0 (22.0, 27.0)	23.0 (21.0, 26.0)	<0.001

* *p*-value for differences in the distribution of sex by protocol: 0.695; *p*-value for differences in the distribution of age by protocol: 0.512; *p*-value for differences in the distribution of BMI by protocol: 0.118.

**Table 6:** Distribution of objective analysis values by protocol.

Variable	Standard Dose	Low Dose
	*n* = 107 (41.80%)	*n* = 149 (58.20%)
Mean Attenuation Lung—*Median (IQR)*	−501.50 (−511.00, −490.50)	−503.00 (−519.00, −486.50)
Mean Image Noise Lung—*Mean (SD)*	76.09 (17.93)	88.99 (24.34)
Average SNR Lung—*Median (IQR)*	6.63 (5.62, 8.11)	5.85 (4.94, 7.25)
CNR Lung—*Median (IQR)*	7.96 (6.85, 9.92)	6.95 (5.38, 8.84)

**Table 7:** Distribution of objective analysis values by estimated difference between protocols (low dose—standard dose) based on median regression models for clustered data (except for Mean Image Noise: linear mixed models).

Variable	Difference (95% CI)	*p*-Value	Adj. Difference (95% CI)	Adj. *p*-Value
Mean Attenuation Lung	−1.5 (−8.1, 5.1)	0.656	−2.7 (−9.8, 4.4)	0.457
Mean Image Noise Lung	12.5 (7.1, 18.0)	<0.001	11.8 (6.2, 17.3)	<0.001
Average SNR Lung	−0.8 (−1.4, −0.2)	0.013	−0.7 (−1.3, -0.0)	0.046
CNR Lung	−1.0 (−1.8, −0.2)	0.015	−0.8 (−1.7, 0.1)	0.083

**Table 8:** Distribution of subjective analysis by examiner #2 (RG2-Radiographer) results by protocol.

Variable	Standard Dose	Low Dose
	*n* = 107 (41.80%)	*n* = 149 (58.20%)
VGA Subjective Image Noise Lung RG2		
*– Moderate*	17 (15.89%)	82 (55.03%)
*– Minimal*	90 (84.11%)	67 (44.97%)
VGA Subjective Image Quality Lung RG2		
*– Acceptable*	15 (14.02%)	31 (20.81%)
*– Excellent*	92 (85.98%)	118 (79.19%)
Artifacts IR RG2		
*– Minor*	25 (23.36%)	63 (42.28%)
*– Not affecting diagnosis*	82 (76.64%)	86 (57.72%)

**Table 9:** Distribution of subjective analysis by examiner #2 (RG2-Radiographer) by estimated Odds Ratios for better results (low dose versus standard dose) based on mixed ordinal logistic regression models.

Variable	Odds Ratio (95% CI)	*p*-Value	Adj. Odds Ratio (95% CI)	Adj. *p*-Value
VGA Subjective Image Noise Lung	0.15 (0.08, 0.28)	<0.001	0.17 (0.08, 0.34)	<0.001
VGA Subjective Image Quality Lung	0.60 (0.29, 1.24)	0.168	0.63 (0.30, 1.33)	0.222
Artifacts IR	0.42 (0.24, 0.72)	0.002	0.38 (0.21, 0.68)	0.001

**Table 10:** Distribution of radiologic findings (by examiner #1) results by protocol (n and % refer only to positive results).

Variable	Standard Dose	Low Dose
	*n* = 107 (41.80%)	*n* = 149 (58.20%)
Consolidation RD1		
*– No*	58 (54.21%)	108 (72.48%)
*– Yes*	49 (45.79%)	41 (27.52%)
Ground Glass Opacity RD1		
*– No*	19 (17.76%)	53 (35.57%)
*– Yes*	88 (82.24%)	96 (64.43%)
Nodules (≥3 mm) RD1		
*– No*	32 (29.91%)	67 (44.97%)
*– Yes*	75 (70.09%)	82 (55.03%)
Cavitation in nodule(s) RD1		
*– No*	102 (95.33%)	139 (93.29%)
*– Yes*	5 (4.67%)	10 (6.71%)
GG halo in nodule(s) RD1		
*– No*	97 (90.65%)	144 (96.64%)
*– Yes*	10 (9.35%)	5 (3.36%)
Diffuse Interlobular septal thickening RD1		
*– No*	71 (66.36%)	104 (69.80%)
*– Yes*	36 (33.64%)	45 (30.20%)
Pleural effusion RD1		
*– No*	59 (55.14%)	83 (55.70%)
*– Yes*	48 (44.86%)	66 (44.30%)
Pericardial effusion RD1		
*– No*	96 (89.72%)	132 (88.59%)
*– Yes*	11 (10.28%)	17 (11.41%)
Lymphadenopathy RD1		
*– No*	74 (69.16%)	103 (69.13%)
*– Yes*	33 (30.84%)	46 (30.87%)

**Table 11:** Distribution of radiologic findings (by examiner #1) by estimated Odds Ratios for positive results (low dose versus standard dose) based on mixed logistic regression models.

Variable	Odds Ratio (95% CI)	*p*-Value	Adj. Odds Ratio (95% CI)	Adj. *p*-Value
Consolidation	0.34 (0.16, 0.74)	0.006	0.32 (0.15, 0.71)	0.005
Ground Glass Opacity	0.30 (0.13, 0.68)	0.004	0.31 (0.13, 0.71)	0.006
Nodules (≥3 mm)	0.50 (0.25, 1.02)	0.056	0.50 (0.24, 1.04)	0.063
Cavitation in nodule(s)	1.34 (0.35, 5.16)	0.674	1.28 (0.31, 5.22)	0.735
GG halo in nodule(s)	0.28 (0.07, 1.06)	0.062	0.30 (0.07, 1.33)	0.114
Diffuse Interlobular septal thickening	0.57 (0.22, 1.51)	0.258	0.69 (0.27, 1.76)	0.436
Pleural effusion	1.04 (0.51, 2.12)	0.916	1.04 (0.49, 2.22)	0.911
Pericardial effusion	3.57 (0.18, 70.48)	0.403	3.32 (0.27, 41.28)	0.351
Lymphadenopathy	0.42 (0.11, 1.61)	0.205	0.45 (0.13, 1.59)	0.215

## Data Availability

The data presented in this study are available in this article. Further inquiries can be directed to the corresponding author.
